# 11.P. Workshop: Tobacco control policy strategies to contribute to the EU’s tobacco-free generation goal

**DOI:** 10.1093/eurpub/ckac129.752

**Published:** 2022-10-25

**Authors:** Organised by: Tilburg University, Amsterdam UMC, University of Amsterdam (Netherlands), Chair persons: Andrea Rozema (Netherlands), Mirte Kuipers (Netherlands)

## Abstract

In 2021, the European Union's (EU) ‘Europe's Beating Cancer Plan’ set the goal to achieve a tobacco-free generation in Europe by 2040, which means that less than 5% of people will use tobacco. The EU proposed policies to address internationally relevant issues such as tobacco taxation, cross-border purchases, packaging, flavours, and online advertising. However, many tobacco control policies need to be adopted and implemented at the national or local level. It is therefore important to exchange research findings and insights on national tobacco control policies. In this workshop we present results mainly from the Netherlands. With a 21% smoking prevalence and scores 53 out of 100 points on the Tobacco Control Scale, it may therefore be considered an average European country in terms of its tobacco control. However, the Netherlands has progressed its tobacco control policies in recent years, in part due to the 2018 National Prevention Agreement. This Agreement included the same goal as the European tobacco-free generation goal. Achieving a tobacco-free generation requires the prevention of smoking initiation among young people, but also smoking cessation among those who already smoke. This is especially important among lower socioeconomic groups, as the smoking prevalence in these groups is higher both in adults and adolescents. Therefore, smoking and tobacco products need to be less visible and available in the environment, and easily accessible smoking cessation support needs to be provided. With this workshop, we cluster very recent evidence on the EU's tobacco-free generation goal. We will present evidence on four policy strategies for the smoke-free generation, in which their potential impact and challenges in implementation will be discussed. These presentations will be followed by a plenary discussion on the implications and the relevance of the four strategies taking into account differences between international settings.

The presentations will focus on four policy areas:

• Policies to reduce smoke-exposure in and around sport clubs

• Policies to reduce availability of tobacco products in the retail environment

• Policies to reduce smoke-exposure in hospitality venues and homes

• Policies to increase access to smoking cessation

The objectives of the workshop are:

1. Present evidence of four tobacco control policies strategies

2. Discussion of the international relevance and implications of the results

**Key messages:**

• We present on multiple settings that can be further capitalised on to achieve a non-smoking norm, including sports clubs, retail outlets, hospitality venues, and the home environment.

• The majority of European countries currently does not have strong tobacco control policies in the presented settings, and the potential of such policies throughout Europe will be discussed.

